# Knowledge‐based deep residual U‐Net (DRU) for synthetic CT generation using a single MR volume for frameless radiosurgery

**DOI:** 10.1002/acm2.70343

**Published:** 2025-12-29

**Authors:** Xiwen Shu, Ke Lu, Jingtong Zhao, John Ginn, Yongbok Kim, Zhenyu Yang, Justus Adamson, Trey Mullikin, Chunhao Wang

**Affiliations:** ^1^ Department of Radiation Oncology Duke University Durham North Carolina USA; ^2^ Medical Physics Graduate Program Duke Kunshan University Kunshan Jiangsu China

**Keywords:** deep learning, frameless SRS, MRI‐only radiotherapy, Residual U‐Net, treatment planning

## Abstract

**Purpose:**

To develop a knowledge‐based deep model for *sCT* generation from a single MR volume in LINAC‐based frameless SRS, enabling the MR‐only workflow without extra CT simulation.

**Methods:**

A total of 139 patients were included in the study, with 120 used for training and 19 for testing. A Deep Residual U‐Net(DRU) was developed to generate *sCT* from patient‐specific high‐resolution T1 + Contrast MR volume, complemented by a healthy brain CT volume from the Visible Human Project that provides CT‐specific anatomical knowledge. To simulate treatment conditions, a template immobilization mask was deformed to align with the patient‐specific *sCT* anatomy, thereby creating a full *sCT_F_* volume. Four metrics, including PSNR, SSIM, RMSE, and MAE were derived to evaluate Hounsfield units(HU) accuracy of *sCT* compared to the ground‐truth CT without immobilization masks. Single isocenter multi‐target SRS plans developed with volumetric modulated arc therapy (VMAT) technique were recalculated within *sCT_F_* volumes to produce simulated dose distributions, which were compared with clinical plan dose distributions using the mean dose difference in the planning target volume(PTV) and gamma index evaluation.

**Results:**

In the test set, the generated *sCT* (statistics are reported as mean ± standard deviation) achieved a PSNR(Peak Signal‐to‐Noise Ratio) of 75 ± 4 dB, Structural Similarity Index (SSIM) of 0.99 ± 0.01, root mean square error (RMSE) of 11.9 ± 5.8 HU, and mean average error (MAE) of 1.4 ± 0.8 HU for brain tissues. When comparing *sCT_F_* dose calculation results against the original plans, gamma index passing rates were 95.8 ± 4.2% for the entire volume and 84.4 ± 15.0% within PTVs, using 3%/1 mm/15% threshold criteria. The median/ interquartile range of PTV dose differences were –2.0% and 2.3%, with all discrepancies below –5.0%.

**Conclusion:**

This study successfully demonstrated the generation and validation of *sCT* images from single‐modality MRI using a knowledge‐based deep model. The results confirm that single‐modality MRI without simulation CT scan effectively supports frameless SRS planning and integrates seamlessly into current clinical workflows.

## 1 INTRODUCTION

Stereotactic radiosurgery (SRS) is an advanced, non‐invasive radiation therapy technique that precisely targets intracranial lesions using highly focused beams to deliver high radiation dose to tumors with sub‐millimeter accuracy, minimizing damage to surrounding healthy tissue while achieving excellent local control.^1^ A critical step in SRS planning involves the integration of high‐resolution MRI for target delineation and CT images for electron density mapping and dose calculation.^2^ However, the current workflow requires separate MR acquisition and CT simulation, followed by image co‐registration between MRI and CT images for treatment planning. A practical challenge of this practice is the additional waiting time between MR acquisition and CT simulation, which can be a logistical problem for patients who may have limited access to frequent hospital visits. This potential waiting time could be more impactful on some brain metastasis (BM) patients, who often require urgent treatment due to rapid tumor growth and may have limited mobility. Additionally, MR‐CT co‐registration may introduce residual errors. With the increasing adoption of frameless SRS, which eliminates the need for rigid head fixation using invasive frames and instead relies on mask‐based immobilization and image guidance, precise image registration becomes even more critical. Misalignment due to registration inaccuracies can introduce setup errors, potentially compromising treatment accuracy. As such, there is a growing interest in an MR‐only workflow for LINAC‐based SRS. This approach could simplify the workflow for SRS planning, especially in frameless SRS applications, and also reduce radiation exposure to patients by eliminating the CT scan.^3^

Synthetic CT (sCT) generation from MRI has emerged as a promising solution that eliminates the need for CT simulation, offering a streamlined and patient‐friendly approach to SRS planning. Conventional methods for sCT generation often rely on multi‐modal MRI sequences, such as T1‐weighted, T2‐weighted, or ultra‐short echo time imaging, to approximate bone anatomy and tissue density.^4^, ^5^, ^6^, ^7^ Although these techniques have shown encouraging preliminary results, they come with drawbacks, including extended scan times, increased complexity in imaging protocols, and variability in image quality across different MRI systems.^8^ These factors restrict their widespread clinical adoption, particularly in time‐sensitive scenarios such as treating brain metastases. In recent years, deep learning approaches, particularly convolutional neural networks (CNNs), have demonstrated significant potential in generating high‐quality sCT images from MRI data.^9^, ^10^, ^11^, ^12^, ^13^ These methods utilize large datasets to learn the complex nonlinear mapping between MRI intensity values and CT Hounsfield units (HUs), which are the standard units used in CT images for electron density information. Among the various CNN architectures, the U‐Net has emerged as a particularly powerful model for medical image‐to‐image translation tasks, including sCT generation.^11^, ^14^, ^15^ Originally developed for biomedical image segmentation, the U‐Net features a symmetric encoder‐decoder structure with skip connections that bridge the two pathways. The encoder extracts hierarchical features from the input image, while the decoder reconstructs the output image at the same resolution as the input. The skip connections allow the model to preserve fine‐grained spatial details, making it highly effective for tasks requiring precise anatomical accuracy.^11^ Studies have shown that U‐Net‐based models can effectively model the relationship between MRI and CT data, producing sCTs that are clinically useful for dose calculation and treatment planning.^9^, ^16^

Despite their promise, many existing deep learning models struggle to accurately capture CT‐specific anatomical details. When trained solely on patient‐specific data, these models may often fail to generalize across diverse anatomical variations, further limiting their clinical applicability.^12^, ^17^ Patient‐specific training often requires a large amount of paired MRI‐CT data, which can be difficult to acquire due to logistical and ethical constraints.^18^ Additionally, variations in patient anatomy, imaging protocols, and scanner differences can lead to overfitting, reducing the model's ability to perform well on unseen data.^19^ To address these limitations, researchers have been exploring techniques that incorporate prior anatomical knowledge or hybrid approaches by combining deep learning with traditional methods. For instance, atlas‐based methods leverage a library of pre‐existing CT scans to enhance model accuracy and improve generalizability.^20^, ^21^ By incorporating anatomical priors, atlas‐based techniques can help mitigate the limitations of purely data‐driven models, particularly in cases with limited training data. A more practical limitation is the absence of patient‐specific immobilization devices (i.e., thermoplastic masks) at MR simulation, primarily due to size incompatibility.^22^ These devices are critical to immobilize patients to ensure precise SRS. Given that certain immobilization masks exhibit noticeable radiation attenuation, accounting for their effects in sCT generation is essential for accurate SRS planning.^23^

Building on these advancements, we propose a knowledge‐based deep residual U‐Net (DRU) for sCT generation using a single high‐resolution T1+C MR volume. A healthy brain CT volume from the Visible Human Project^24^ serves as a template, integrating anatomical knowledge of bone and tissue structures. To simulate real‐world SRS conditions, a template immobilization mask is integrated into the sCT generation process to form a patient‐specific one with deformable image registration. The whole workflow is evaluated in two key areas: (1) HU‐based image accuracy, by comparing the derived *sCT* to the ground‐truth CT at a pixel‐wise level, and (2) dosimetric fidelity, by assessing the clinical plan calculated on the derived *sCT* volume against the original clinical plan.

## 2 MATERIALS AND METHODS

### 2.1 Patient data

This study was approved by our institutional review board and retrospectively analyzed data from 139 patients treated with single‐isocenter multi‐target SRS. A summary of patient information can be found in the Supporting Information. All SRS plans were developed with VMAT technique using 6 MV flattening filter‐free (FFF) photon beam with Varian TrueBeam STx (Varian Medical System, Palo Alto, CA) medical linear accelerators (LINACs). The dataset was divided into training (120 patients) and testing (19 patients) sets, with no overlap between the groups. Within the training set, 20 patients (1/6) were reserved for the validation purposes. Each patient underwent separate CT and MR simulations. CT simulation was performed using a BrainLab (BrainLab, Munich, Germany) frameless thermoplastic mask with a 400 mm in‐plane field‐of‐view (FOV) with 512 × 512 matrix size and 1 mm slice thickness. MR simulation was conducted on a dedicated Siemens (Siemens Healthineers, Erlangen, Germany) Skyra 3T simulator. For target delineation purposes, the high‐resolution T1 contrast‐enhanced (T1+c) scans were acquired using the spoiled gradient echo (SPGR) technique with a 1 mm isotropic voxel size. A summary of detailed imaging parameters can be found in the Supporting Information. The T1+c volumes were then resampled to match the CT image grid for subsequent deep learning tasks.

### 2.2 Overall study design

**FIGURE 1:**
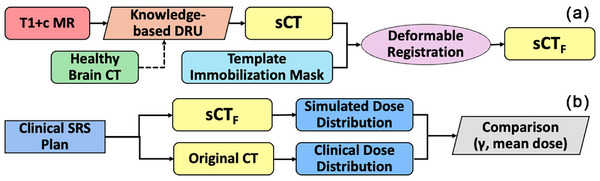
Workflow summarizing key designs in this work.

Figure 1 summarizes the key technical designs in this work. The workflow consists of two primary components: (A) the generation of *sCT* images and (B) the comparison of dose distributions for validation. The first stage (A) summarizes the *sCT* generation. The process begins with the acquisition of T1+c MR images, which serve as input for the designed DRU. This DRU generates an initial *sCT* by leveraging a healthy brain CT volume from Visible Human Project^23^ as the knowledge base. A template immobilization mask is then incorporated into the workflow by a deformable image registration process to produce a full synthetic CT (*sCT_F_*), as the final product image. The second stage (B) focuses on validating the accuracy of *sCT_F_*. For each test case, the original clinical SRS treatment plan is applied to the *sCT_F_* to generate a simulated dose distribution, and this distribution is compared to the one from the clinical treatment plan as the ground truth using the gamma index (γ) analysis.

### 2.3 Deep residual network

**FIGURE 2:**
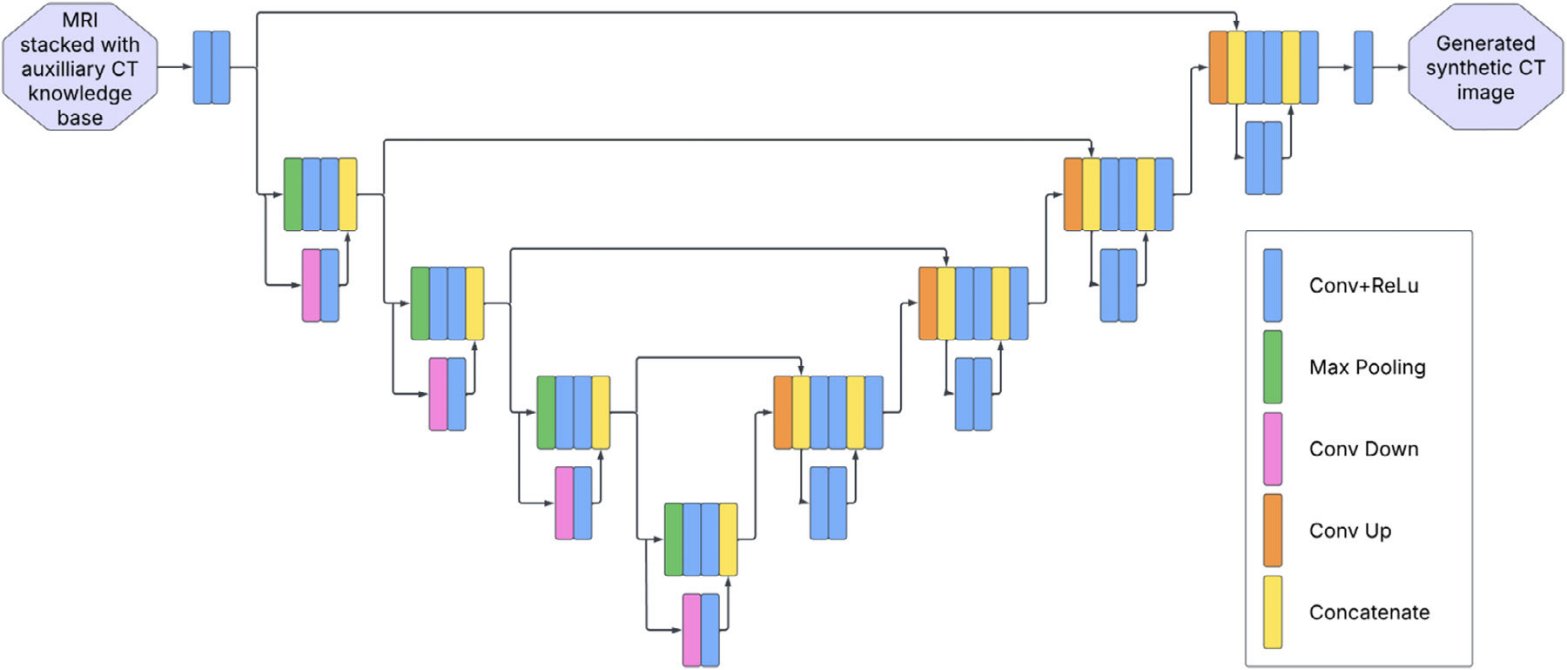
Network structures of the proposed DRU. DRU, deep residual U‐Net.

Figure 2 illustrates the architecture of the proposed DRU, which is based on the 3D U‐Net framework—a widely adopted CNN for medical image segmentation and synthesis. DRU features a symmetric encoder‐decoder structure with residual connections to improve feature learning and gradient flow.^11^ The DRU takes a 3D volume as the input, which is implemented by appending the axial MR slice at the location of interest as an additional index along the 3rd dimension of the knowledge base CT volume. The DRU output is a single axial *sCT* slice corresponding to the anatomy of the axial MR slice within the 3D input. The encoder uses convolution and max pooling layers to extract hierarchical, multi‐scale features from MRI, while the decoder employs transposed convolutions to reconstruct spatial resolution. Skip connections between encoder and decoder layers help retain fine anatomical details, such as edges and textures, ensuring accurate cross‐modality mapping between MRI and CT domains. At the core of the DRU architecture are the residual connections, which are integrated into both the encoder and decoder pathways.^12^ These connections add the input of a block to its output, creating shortcuts that enable the network to efficiently learn residual mappings. The residual connections enable the model to focus on learning the differences between MRI and CT domains, rather than reconstructing the entire image from scratch. The final output block of the DRU consists of a convolutional layer followed by a linear activation function, which reconstructs the final *sCT* image.

Prior to the DRU execution, MR intensity values were normalized to the [0,1] range based on the maximum value within each 3D volume. Similarly, the healthy brain CT volume, after resampling to the planning CT grid size, was normalized to the [0, 1] range with an assumed 12‐bit acquisition setting. To achieve optimal performance, a hyperparameter optimization process was conducted during the model training phase. Various combinations of hyperparameters were evaluated, including different numbers of epochs (20, 100, 150, and 200), batch sizes (4, 8, and 16), and learning rates (0.01, 0.1, 0.5, and 0.001). These combinations were tested to identify the configuration that maximized model accuracy and convergence efficiency. The optimal hyperparameter combination was determined to be 150 epochs, a batch size of 4, and a learning rate of 0.01, which delivered the best balance of training stability and validation accuracy. The whole training process was implemented within a PyTorch (1.12.1) environment on a dedicated workstation with 1x Intel Xeon CPU @ 4.10 GHz, 2x Nvidia A6000 GPU, and 128GB usable RAM. Each training session took approximately 10 h to complete.

### 2.4 Immobilization mask deformation

**FIGURE 3:**
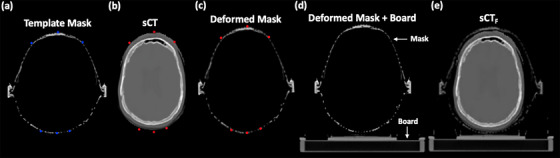
An example of immobilization mask deformation process.

A head phantom CT volume with BrainLab (BrainLab, Munich, Germany) frameless thermoplastic mask was acquired, and a mask template image was generated by subtracting the head phantom from this volume. The template image (Figure 3d) was then divided into two components: 1) the thermoplastic mask portion that consists of an anterior face section and a posterior support section; and 2) the frameless board portion that includes the LINAC couch attachment board and two spacer bars (one on the left, one on the right) to hold the two mask sections together.

The mask deformation process focused on adapting the thermoplastic mask portion to the derived *sCT*. We utilized an open‐source image deformation program with a graphical user interface (GUI) to implement the proposed deformation process.^25^ Developed in PyCharm, this program allows users to select paired anchor points in the target and source image volumes, which serve as fixed alignment references. In this study, as shown in Figure 3a, the template mask was initialized with anchor points marked in blue along key anatomical regions where the thermoplastic mask typically contacts the patient, including the lateral edges of the face, the nose tip, and the posterior‐anterior neck region near the skull base. The remaining deformation field is then derived using spline interpolation and is modified for full 3D implementation. Figure 3c demonstrates the resulting deformed mask, with red points identically positioned to those in the *sCT*. Subsequently, the frameless board portion was rigidly registered to the mask portion by aligning the spacer bars with the mask connection level (deformed mask + board) in Figure 3d. To ensure proper alignment with the horizontal surface, slight adjustments were made to the spacer bars to accommodate head tilt if necessary. All deformation results were carefully reviewed by experienced medical physicists to ensure a proper and clinically reasonable fit.

### 2.5 Evaluation

#### 2.5.1 HU comparison in sCT

To evaluate the *sCT* accuracy in terms of HU representation, four quantitative image similarity metrics were employed in 3D. First, peak signal‐to‐noise ratio (PSNR) was used to measure the overall image quality by quantifying the ratio between the maximum signal power and the corruption noise present in the *sCT*. PSNR is defined as:

(1) $$PSNR=10\times log_{10}\left(\frac{MAX_{I}^{2}}{MSE}\right)$$

where *MAX_I_* represents the maximum possible pixel intensity value (e.g., 4096 for 12‐bit CT images) and *MSE* is the mean squared error between the *sCT* ($I_{sCT}$) and the reference CT ($I_{CT}$). Higher PSNR values indicate greater fidelity of the *sCT* relative to the reference CT, as they reflect a lower level of noise and distortion in the generated image. This metric is particularly useful for assessing the overall quality of the *sCT* in terms of pixel intensity accuracy.

Second, structural similarity index measure (SSIM) was calculated to assess the degree of structural preservation between the *sCT* and the reference CT, incorporating factors such as luminance, contrast, and structural details. SSIM is computed as:

(2) $$SSIM\left(x,y\right)=\frac{\left(2\mu_{x}\mu_{y}+C_{1}\right)\left(2\sigma_{xy}+C_{2}\right)}{\left(\mu_{x}^{2}+\mu_{y}^{2}+C_{1}\right)\left(\sigma_{x}^{2}+\sigma_{y}^{2}+C_{2}\right)}$$

where $\mu_{x}$ and $\mu_{y}$ are the local means, $\sigma_{x}$​ and $\sigma_{y}$ are the variances, $\sigma_{xy}$ is the covariance of the *sCT* and reference CT, and $C_{1}$​ (0.01^2^) and $C_{2}$​ (0.03^2^) are constants to stabilize the division. SSIM values range from –1 to 1, with a value closer to 1 indicating a higher degree of similarity between the two datasets. This metric is particularly effective for evaluating the preservation of anatomical structures and fine details in the *sCT*.

Third, root mean square error (RMSE) was computed to quantify the standard deviation of the residual differences between the *sCT* and the reference CT. RMSE is defined as:

(3) $$RMSE=\sqrt{\frac{1}{N}\sum_{i=1}^{N}{\left(I_{sCT}\left(i\right)-I_{CT}\left(i\right)\right)}^{2}}$$

where *N* is the total number of pixels, and $I_{sCT}\left(i\right)$ and $I_{CT}\left(i\right)$ represent the pixel intensities of the *sCT* and reference CT respectively. Lower RMSE values signify better image agreement. RMSE is a widely used metric for assessing the accuracy of image synthesis tasks, as it provides a direct measure of the magnitude of errors.

Lastly, mean absolute error (MAE) was derived to capture the absolute intensity differences between the two datasets, with lower values reflecting improved accuracy. MAE is defined as:

(4) $$MAE=\frac{1}{N}\sum_{i=1}^{N}\left|I_{sCT}\left(i\right)-I_{CT}\left(i\right)\right|$$

where *N* is the total number of pixels. Lower MAE values reflect improved accuracy, as they indicate smaller average deviations in pixel intensity between the *sCT* and the reference CT. Unlike RMSE, MAE is less sensitive to outliers, making it a robust metric for evaluating the overall intensity accuracy.

#### 2.5.2 Dose calculation accuracy in sCT_F_

The accuracy of the dose calculations between the original plan and *sCT* plan was first assessed by comparing the mean dose difference within the PTV. At our institution, a mean dose difference of less than 5% is considered acceptable during independent secondary dose calculation check. PTV mean dose difference check ensures that the *sCT_F_* does not introduce large dosimetric errors. In addition to the PTV mean dose comparison, gamma index evaluation was performed to quantify the spatial and dosimetric agreement between two plans.^26^ We used a protocol with a 3% dose difference, 1 mm distance‐to‐agreement (DTA) tolerance, and a 15% dose threshold for gamma index analysis, consistent with our clinical implementation criteria. Clinically, a gamma passing rate of 90% within the BODY is considered acceptable. However, we do not enforce a gamma passing rate threshold within the PTV, as it is highly influenced by the potentially small PTV size.

## 3 RESULTS

**FIGURE 4:**
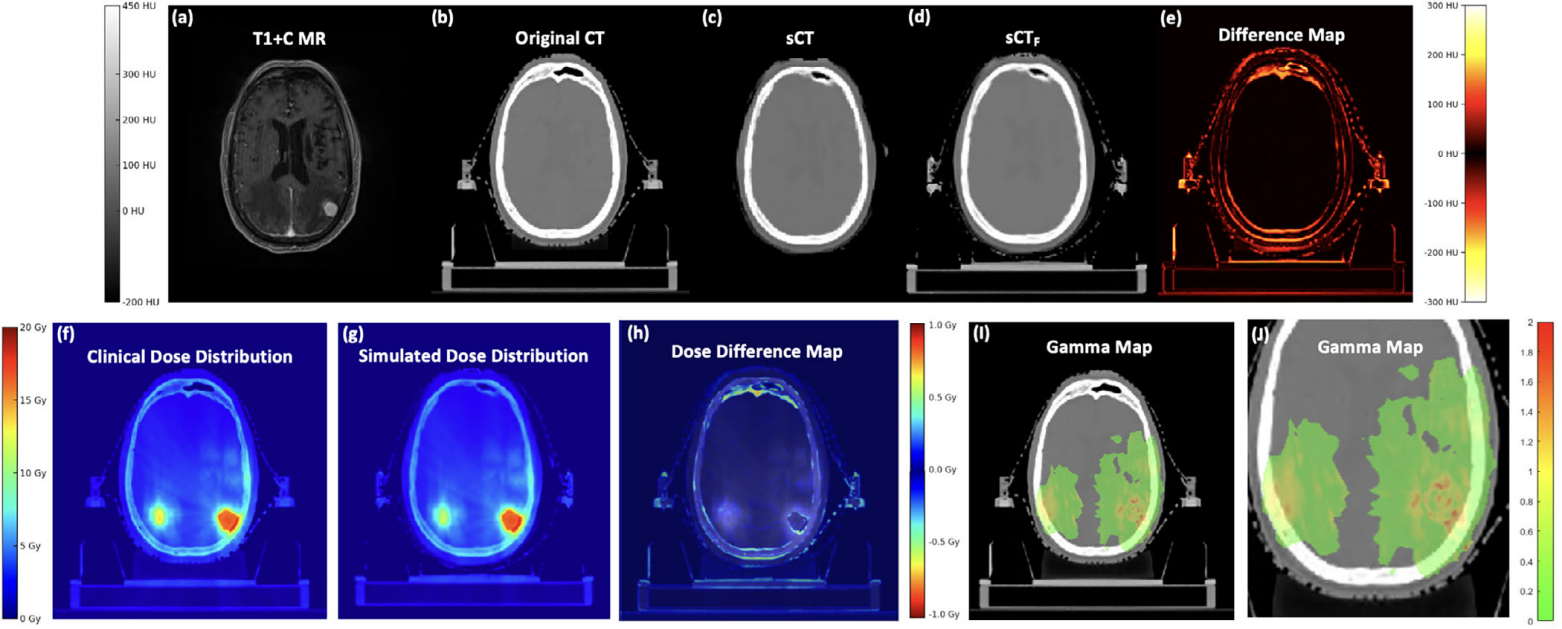
An example case of *sCT* and *sCT_F_*, including comparisons with original CT, dose distribution comparisons, and Gamma analysis.

Figure 4 illustrates an example case of *sCT_F_* generation process and dose evaluation. The T1+c MR image (Figure 4a) shows cranial anatomy with more details. Figure 4b is the original CT scan, which serves as the ground truth. Figure 4c is the initial *sCT* generated from the MR data. This *sCT* closely resembles the original CT in terms of tissue contrast and structural details, but minor deviations can be observed in the skull and skull–brain interface (Figure 4e). The final image *sCT_F_* represents the refined synthetic CT after immobilization mask integration. The good agreement between *sCT_F_* and the reference CT suggests the effectiveness of immobilization mask deformation design, further enhancing the usability of the *sCT_F_* for SRS in a clinical setting.

### 3.1 HU comparison in sCT

A quantitative evaluation of the *sCT* image quality within the test set is summarized in Table 1, where PSNR, SSIM, RMSE, and MAE (reported as mean value ± standard deviation) were computed across different anatomical regions, including the whole body, bone, and brain tissues. The results indicate a strong agreement between the *sCT* and the reference CT, with particularly high accuracy in the brain region. The brain‐specific analysis yielded a PSNR of 75 ± 4 dB and an SSIM of 0.99 ± 0.002, demonstrating a high level of image fidelity and structural preservation. RMSE and MAE values for the brain were also the lowest among the three evaluated regions, at 11.9 ± 5.8 HU and 1.4 ± 0.8 HU, respectively, confirming that the model achieves precise HU estimations in both high‐contrast and low‐contrast structures. Comparatively, the bone region (i.e., the skull) exhibited slightly lower PSNR and higher RMSE with values of 64 ± 2 dB and 35.0 ± 8.3 HU respectively, which reflects the inherent challenge of modeling high‐density structures. The BODY (i.e., the entire tissue region used for dose calculation) assessment is slightly lower than the localized tissue evaluations but still indicates a high overall agreement across the entire anatomical structure. The decreasing trend in RMSE and MAE from the whole body to the brain highlights the improved accuracy of *sCT* generation in soft tissues, particularly in brain regions where precise HU estimations are critical for radiotherapy applications.

**TABLE 1:** PSNR, SSIM, RMSE, and MAE results of sCT for BODY, bone, and brain. Statistics are reported as mean value ± standard deviation.

	PSNR (dB)	SSIM	RMSE (HU)	MAE (HU)
BODY	59 ± 2	0.98 ± 0.005	57.7 ± 10.2	10.2 ± 2.4
Bone	64 ± 2	0.99 ± 0.001	35.0 ± 8.3	2.7 ± 1.0
Brain	75 ± 4	0.99 ± 0.002	11.9 ± 5.8	1.4 ± 0.8

Abbreviations: MAE, mean average error; PSNR, peak signal‐to‐noise ratio; RMSE, root mean square error; SSIM, structural similarity index.

The second row of Figure 4 presents the dosimetric evaluation of *sCT_F_*. Figure 4f displays the clinical dose distribution as the ground truth. Figure 4g shows the simulated dose distribution, which is recalculated using the *sCT_F_*. The distribution closely resembles the ground truth as indicated by the dose difference map in Figure 4h, which indicates that the *sCT_F_* preserves the essential radiological properties required for accurate dose calculations. Figure 4i,j (zoomed view) presents the gamma map. The gamma analysis demonstrates a high degree of concordance, as indicated by the predominance of green regions (*γ*≤1), which signifies an acceptable level of agreement within clinically established thresholds. A few isolated areas exhibit deviations represented by yellow to red pixels (*γ* > 1) suggesting localized discrepancies.

### 3.2 Dose calculation accuracy in sCT_F_

Quantitative dosimetric evaluation demonstrated the gamma index passing rate of all 19 test patients was found to be 95.8 ± 4.2% for the whole‐body region and 84.4 ± 15.0% within the PTVs. The high gamma passing rate for the whole body suggests that the *sCT* is well‐suited for overall dose calculation, while the slightly lower agreement within PTVs reflects the potentially greater sensitivity of high‐dose gradient regions to potential variations in HU estimation and anatomical alignment. Overall, PTV mean dose results in the simulated dose distribution were lower than the clinical plan results (see Figure S1 in Supporting Information). The mean dose difference (–2.3%) and median dose difference (–2.0%) remained acceptable, with an interquartile range of 2.3%. The largest PTV dose difference was –4.8%. These results indicate the potential systematic PTV underdose using *sCT_F_*; nevertheless, the observed deviations were minimal and unlikely to affect clinical treatment outcomes. Even in the most extreme case (–4.8% PTV mean dose difference), the deviation remained within the secondary clinical evaluation threshold at our institution, which permits up to a 5% difference in PTV mean dose without gamma passing rate cutoff for SIMT (i.e., Single Isocenter Multiple Target) SRS plans. Further analysis revealed a strong correlation between the PTV mean dose difference and the gamma passing rate within the PTV (Pearson correlation coefficient *r* = 0.91, *p* < 0.01). In contrast, although the lowest gamma passing rate (54.7%) was found to be within the smallest PTV (0.15cc), neither the PTV gamma passing rate (*r* = 0.24, *p* = 0.32) nor the PTV mean dose difference (*r* = 0.34, *p* = 0.15) demonstrated any meaningful correlation with PTV volume.

## 4 DISCUSSION

In this study, a novel DRU that effectively generates *sCT* images from a single MR volume for streamlining frameless SRS workflow was developed. It offers a practical alternative to conventional CT simulation with learnt MR‐to‐CT image contrast change. The first major innovation in this study is the development of the DRU, which incorporates a template CT image from the Visible Human Project to enhance CT‐specific anatomical details. Prior work in sCT generation has primarily relied on end‐to‐end deep learning models trained on paired MRI‐CT datasets.^9^ However, most existing methods rely on multi‐modal inputs or specialized MRI sequences, which may not be readily available in clinical settings.^27^ This study uniquely focuses on leveraging a single diagnostic MR volume to generate sCT, addressing a critical gap in the literature by providing a practical and widely applicable solution. While multi‐modal approaches have demonstrated high accuracy, the integration of prior anatomical knowledge in our design enhances single‐modality MR‐based sCT generation. To this end, the DRU incorporates a template CT image refining sCT predictions and improving the overall accuracy.^28^ Our results suggest that high‐resolution T1+C MR imaging alone is sufficient for accurate *sCT* generation when combined with the average anatomy. Prior studies have also explored various deep learning architectures for sCT generation. For instance, a cycle‐GAN‐based approach for multi‐sequence MR‐to‐CT synthesis achieved an SSIM of 0.97 and RMSE of approximately 18 HU.^29^ Similarly, a hybrid U‐Net and GAN model for multi‐sequence MRI‐based sCT generation reported mean HU differences of 12 ‐ 15 HU.^30^ The performance of our model, which relies solely on a single MR sequence, surpasses these prior methods in PSNR (75.40 dB vs. 68–72 dB) and SSIM (0.99 vs. 0.97–0.98). These results support the incorporation of anatomical knowledge from the Visible Human Project to further enhance anatomical detail preservation and reduce HU estimation errors. Additionally, voxel‐based and atlas‐based approaches have reported lower gamma index passing rates (85%) compared to the 95.77 ± 4.17% achieved in our study^31^ further supporting the advantages of deep learning‐based feature extraction over simple registration‐based techniques.

The second innovation is the incorporation of immobilization mask deformation, which enhances the accuracy of *sCT*‐based dose calculations by more accurately reflecting real‐world SRS delivery conditions. Traditional sCT generation methods often overlook the physical effects of immobilization masks, which can introduce dose discrepancies and compromise treatment accuracy. Previous studies have primarily focused on improving sCT accuracy for soft tissue and bone delineation but have not explicitly considered the attenuation effects of immobilization devices.^10^ However, the immobilization devices could introduce dose perturbations of up to 5% in certain clinical scenarios, underscoring the need for their inclusion in treatment planning models.^32^ In this work, we explicitly account for mask‐related attenuation within the *sCT* framework, providing a more realistic representation of treatment conditions. By incorporating this factor, our approach enhances the accuracy of dose calculations and aligns with the broader effort to refine sCT methodologies for high‐precision radiotherapy applications. Furthermore, our methodology builds on the work that highlighted the importance of accurate material characterization in sCT generation, particularly for non‐tissue elements such as immobilization devices.^33^ This is critical as the field moves toward increasingly personalized and precision‐based treatment paradigms, where even small improvements in dose accuracy can translate into better clinical outcomes.

Third, the effectiveness of this *sCT_F_* generation was further validated through dosimetric analysis. VMAT plans were recalculated on *sCT_F_* volumes and compared to clinical treatment plans. The mean PTV dose difference of –2.3 ± 1.5% is consistent with prior studies, such as those using a GAN‐based method, which reported an average dose difference of –3.0%^34^ and –2.1% in a pelvis‐specific cycleGAN approach,^35^ Hybrid methods combining MRI and deformable image registration (DIR) showed larger deviations (–3.5%)^36^ underscoring the relative robustness of our approach. The largest deviation (–4.8%) in our study remains within the clinically acceptable 5% tolerance and compares favorably to outliers reported in other works. Despite these deviations, our overall gamma passing rate of 95.8% for the entire volume is comparable to state‐of‐the‐art results, such as 96.5% global gamma rates reported for head and neck sCT.^7^ This consistency confirms that our model produces dosimetrically robust *sCT* images suitable for clinical use.

**FIGURE 5:**
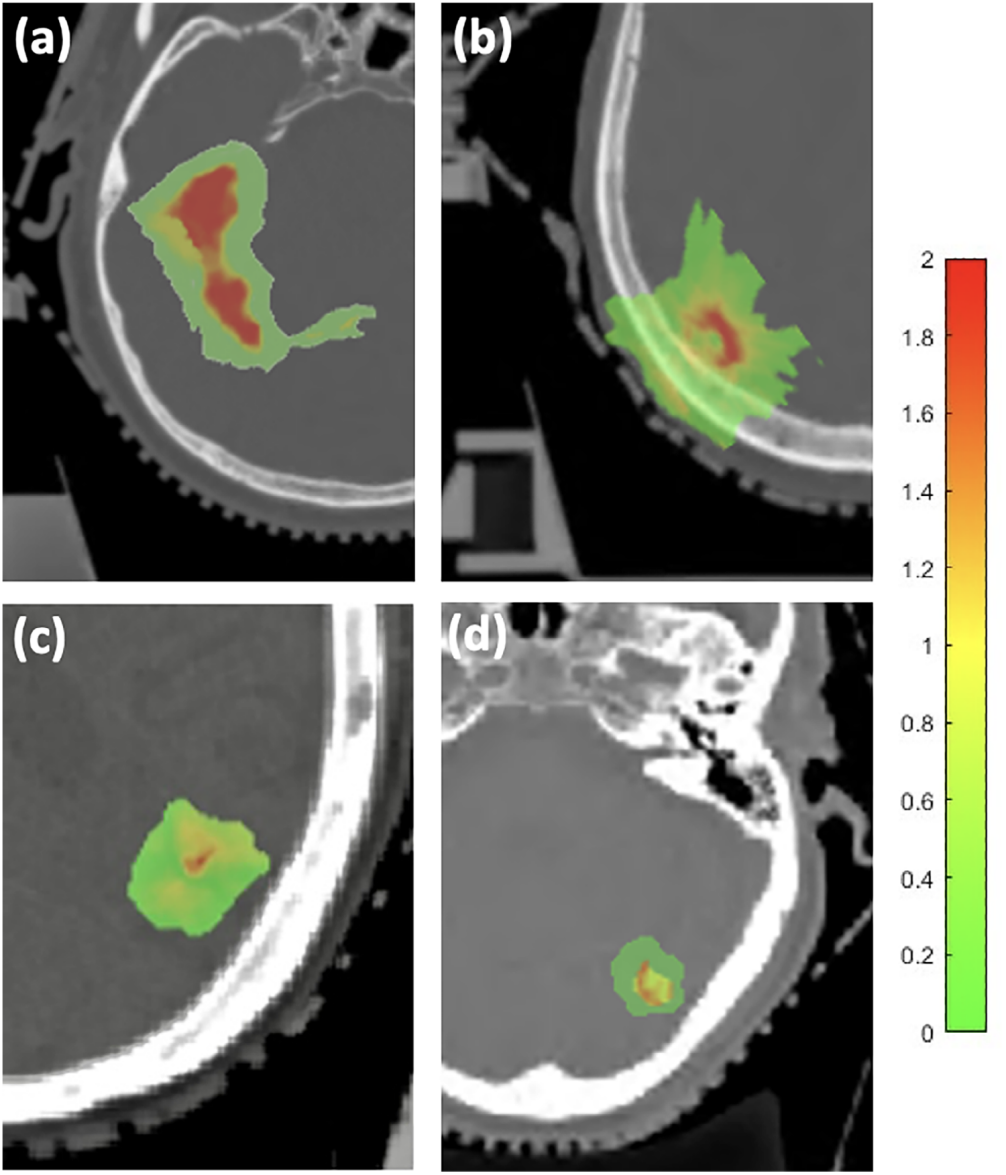
Gamma map results of four additional test patients with zoomed‐in view.

In prior works, dose discrepancies were more pronounced in smaller PTVs. For instance, in prostate radiotherapy, studies have reported gamma passing rates dropping to 85% (2%/2 mm) for small PTVs (< 10 cm^3^) due to HU inaccuracies near tissue interfaces, with localized dose deviations up to 5%.^37^ Similarly, gamma rates as low as 82% were observed for brain SRS targets < 5cc, attributed to misrepresented dose gradients in *sCT*s^38^. Although our findings partially align with this trend, quantitative analysis did not reveal any meaningful correlation between PTV size and either the mean dose difference or the gamma passing rate within the PTV. Figure 5 illustrates zoomed‐in gamma maps for four additional test patients. Figure 5a (gamma passing rate = 64.7%, mean dose difference = –3.6%) and (b) (gamma passing rate = 58.9%, mean dose difference = –4.8%) represents cases with larger PTV dose discrepancy. High gamma values were mainly observed near the center of the dose volume, within or very close to the PTV. In contrast, Figure 5c (gamma passing rate = 93.9%, mean dose difference = –1.9%) and (d) (gamma passing rate = 98.9%, mean dose difference = –2.3%) show cases with more moderate discrepancies. Although these also display similar patterns of high‐gamma voxel distribution, they are visually indistinct from the other cases except in the magnitude of gamma values. Figure 5 and Figure S1 suggests that the dose distribution in *sCT_F_* tends to be systematically offset lower, resulting in more gamma failures in high‐dose regions within or around the PTV. One likely source of error is HU inaccuracy in the skull, supported by higher RMSE and MAE in Table 1 compared to brain tissue, and visible gamma failures in Figures 4i and 5b. Dose discrepancy may also be influenced by beam design factors, such as couch angles and arc ranges. When beam entrance passes through skull regions affected by HU errors, dose calculation accuracy (and thus gamma performance) can be compromised. This is somewhat indicated by Figure 5a, where the target is located near the base of skull. A future quantitative analysis examining PTV location versus dose discrepancy, using a future larger test cohort from our active SRS program, would be valuable. In addition to HU error, positioning inaccuracies of the synthesized immobilization mask system can influence treatment precision. For instance, the longitudinal extent of the frameless mask board affects the vertex beam's dose disposition due to its overlap with the entry path, potentially introducing errors in posterior PTVs. This highlights the clinical importance of modeling mask deformation in planning, with opportunities for further enhancement to improve dosimetric outcomes. PTV dose differences may also relate to the MLC modulation strength in VMAT arcs, a known challenge in small‐field dosimetry. Stronger modulation, reflected by smaller MLC apertures across control points, may be more sensitive to HU inaccuracies. However, such effects could be confounded by PTV size and the number of lesions, which should be considered in the design of quantitative dose sensitivity study. Given the variability in beam design, target distribution, and inverse optimization strategies, it may be premature to isolate a single dominant factor affecting *sCT_F_* dose accuracy using current test patients. An ablation study using a digital phantom—simulating various HU errors, immobilization mask offset, and SIMT SRS plan designs—would be instrumental in addressing this question. This is a key focus of our planned future work to support potential clinical adoption of *sCT_F_*.

Other limitations should also be acknowledged to guide future improvement. To begin with, the current study was conducted on a specific patient cohort undergoing frameless SRS, with a training dataset predominantly composed of patients with brain metastases. Although our model demonstrated strong performance within this context, its generalizability to other radiotherapy scenarios such as fractionated radiotherapy for primary brain tumors remains uncertain. It is essential to further expand the training dataset to include diverse patient populations and anatomical regions as well as conduct multi‐institutional validation studies. As a result, the applicability and scope of the model will be extensive.^8^, ^39^ Next, the current design focused on using a single T1+c MR volume. Although this simple approach is effective, incorporating multi‐modality MRI could provide additional benefits by leveraging different image contrasts for brain soft tissue and skull structures. Complementary information from multiple MR modalities may enhance HU quantification in *sCT*, particularly for brain soft tissue. On the other hand, accurate skull modeling is equally important due to its impact on dose calculation from high HU values. Future studies utilizing multi‐modality MRI would be a promising direction for further improvement. Additionally, the proposed workflow assumes an idealized clinical environment with high‐quality MRI acquisitions and well‐controlled registration processes. In real‐world settings, factors such as patient motion, MR simulator variability, and differences in MRI simulation protocols may introduce variability in sCT generation and subsequent dose calculations. It is critical to investigate how variations of technical parameters of the same MR modality and variations of patient condition during MR acquisition affect sCT results across diverse clinical environments.^34^, ^40^ Collaboration among institutions to establish best practices and guidelines will be beneficial to the successful implementation of this technology.

## 5 CONCLUSION

The application of a knowledge‐based deep learning model for *sCT* generation from an MR volume has demonstrated its effectiveness in streamlining SRS workflows by eliminating the dependency on the CT simulation. The model's adaptability and integration into clinical workflows highlight its potential use for broader applications in radiation oncology, paving the way for more efficient and patient‐centric treatment approaches.

## AUTHOR CONTRIBUTIONS

Xiwen Shu, Ke Lu, and Chunhao Wang conducted the primary research, developed the model, and performed data analysis. Jingtong Zhao and Zhenyu Yang supported data processing and implementation. John Ginn, Trey Mullikin, Justus Adamson, and Yongbok Kim provided clinical insights and context for translational applications. Chunhao Wang supervised the project and guided the research strategy. Xiwen Shu led the manuscript writing. All authors reviewed and approved the final version.

## CONFLICT OF INTEREST STATEMENT

None associated with this work.

## Supporting information



Supporting Information
