# Supplementary material for: Knowledge‐based deep residual U‐Net (DRU) for synthetic CT generation using a single MR volume for frameless radiosurgery
Source: J Appl Clin Med Phys. 2025 Dec 29;27(1):e70343. doi: 10.1002/acm2.70343 (PMC12745906; doi:10.1002/acm2.70343)
Supplement: Supplementary file 1 — Supporting Information [file ACM2-27-e70343-s001.docx]

# Supplementary Document

Table S1. A brief summary of patient information

| **Gender** |  |
| --- | --- |
| Male | 77/139 patients |
| Female | 62/139 patients |
| **Disease Type** |  |
| Brain metastasis | 116/139 patients |
| Recurrent glioma | 18/139 patients |
| Others | 5/139 patients |
| **Lesion Number** |  |
| Minimum | 2 |
| Maximum | 27 |
| 20%-80% Percentiles | 2-9 |
| Median | 4 |
| **Overall PTV size** |  |
| Minimum | 0.15cc |
| Maximum | 81.33cc |
| 20%-80% Percentiles | 0.78cc–14.55cc |
| Median | 4.22cc |
| **Dose fractionation** |  |
| 1 fraction | 79/139 patients |
| 3 fractions | 19/139 patients |
| 5 fractions | 41/139 patients |

Table S2. A summary of T1+C (SPGR) acquisition parameters


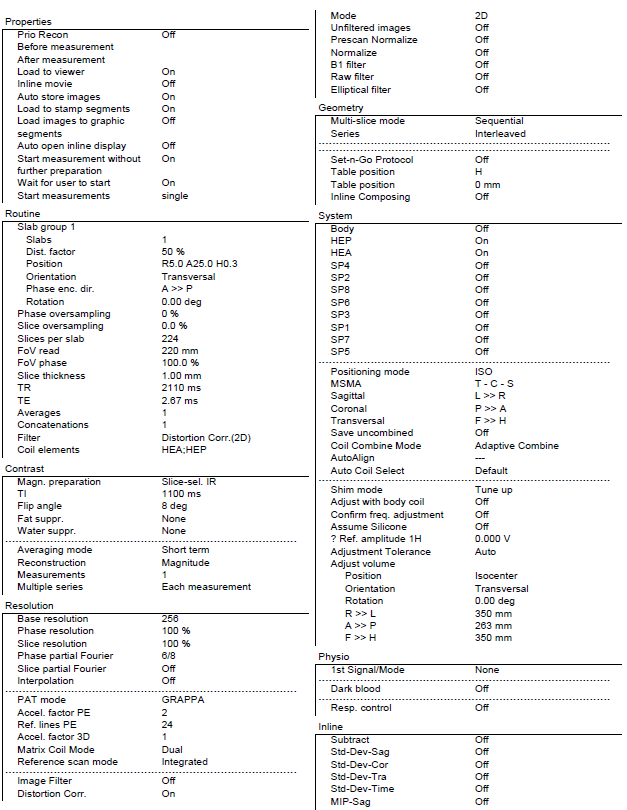


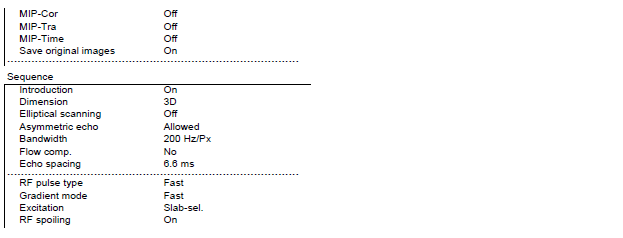


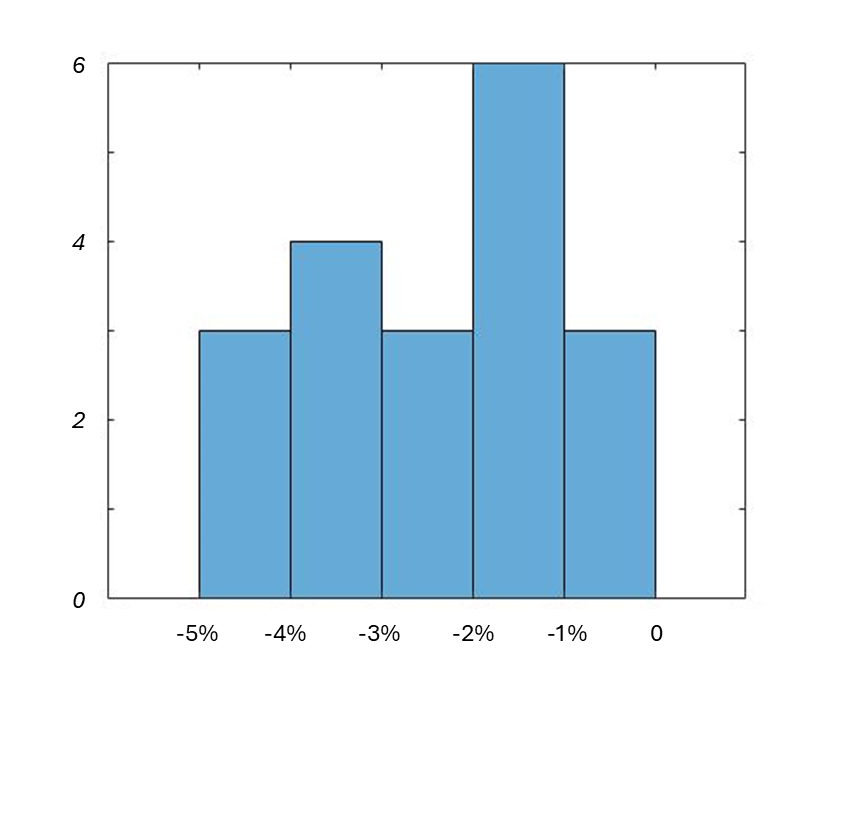


Table S1. PTV mean dose difference histogram of all 19 test patients
